# Optimal position of lipped acetabular liners to improve stability in total hip arthroplasty—an intraoperative in vivo study

**DOI:** 10.1186/s13018-018-1000-1

**Published:** 2018-11-19

**Authors:** Raphael Hau, Joshua Hammeschlag, Christopher Law, Kemble K. Wang

**Affiliations:** 1 Department of Orthopaedic Surgery, Box Hill Hospital, Eastern Health, 8 Arnold Street, Box Hill, Melbourne, VIC 3128 Australia; 2 Department of Orthopaedic Surgery, Northern Health, Melbourne, 185 Cooper St, Epping, Melbourne, VIC 3076 Australia; 3 Monash University, Melbourne, Wellington Road, Clayton, Melbourne, VIC 3800 Australia; 4 Epworth Eastern Hospital, Melbourne, 1 Arnold St, Box Hill, Melbourne, VIC 3128 Australia

**Keywords:** Elevated liner, Instability, Total hip arthroplasty

## Abstract

**Background:**

Lipped or elevated acetabular liners are frequently used in total hip arthroplasty to improve stability. However, the optimal position of the lip is not known. The purpose of this study was to determine the optimal position of lipped acetabular liners in total hip arthroplasty performed with a posterior approach.

**Methods:**

In 14 hips, lipped trial liners were placed intraoperatively in various positions around the posterior clock-face of the implanted acetabular shell component. For each liner position, stability of the hip was tested at maximal hip flexion with gradually increasing internal rotation until subluxation occurred, at which point the position of the hip was measured using smartphone accelerometer-based goniometers. Smartphone goniometers were first validated against a computer-assisted navigation system. Post-operative radiographs were analyzed for cup inclination angle, cup anteversion angle, and femoral offset.

**Results:**

Mean cup inclination angle in our series was 31° ± 6°. The most common liner position that imparted the greatest stability to posterior subluxation was posteriorly and inferiorly (4 o’clock position for left hip, or 8 o’clock position for right hip). The range for most stable liner position for different patients varied from postero-superior (11 o’clock/1 o’clock position) to directly inferior (6 o’clock position). Comparing a non-lipped liner to a lipped liner placed in the optimal position, the average difference in internal rotation gained before dislocation was 23°. There was no association between cup inclination or anteversion angle with liner position of greatest stability.

**Conclusion:**

In hip replacements performed through a posterior approach and with mean cup inclination angle of 31° ± 6°, placing the lip of the elevated liner in the postero-inferior quadrant may impart more stability than in the postero-superior quadrant.

## Introduction

Instability following total hip arthroplasty remains a significant problem and accounts for up to 34% of revisions in major joint registries [1–3]. It is the highest cause of revision in the early postoperative period [1]. Many intraoperative factors contribute to stability and need to be carefully addressed, including optimizing tissue tension, acetabular and femoral component positions, and prosthesis head/neck size ratio [4]. One additional method often employed to improve stability is the use of a lipped or elevated acetabular liner.

While non-lipped liners have the same depth around its entire circumference, lipped liners have an increased height in one segment of the rim. This theoretically increases the jump distance required for the prosthetic femoral head before dislocation can occur in the direction of the elevated rim [5]. Since its introduction by Charnley [6], the use of the lipped liner has increased. In a large series by the Mayo Clinic, lipped liners were used in more than 80% of primary total hip arthroplasties [5]. Although not universally accepted, several large studies have shown that the use of a lipped liner is associated with reduced risk of instability or revision for instability [5, 7–9].

Despite its popularity and apparent usefulness, optimal positioning of the elevated portion of the lipped liner has never been formally established. In hips performed with a posterior approach, the majority of dislocations occur in the posterior direction [5, 10]. It would be logical, therefore, that the elevated part of the liner should be placed in the posterior half of the acetabular shell. However, to the best of our knowledge, there is no evidence in literature to suggest that the postero-superior quadrant is the optimal location as is often traditionally assumed [4, 7, 11]. Clinically and anecdotally, most hip dislocations occur in a position of high hip flexion such as when getting out of a low chair or a low bed. This seems to be supported by biomechanical studies showing that high hip flexion is the primary requirement for posterior dislocation [12–14]. Similarly, the most commonly relied -on maneuver intraoperatively in testing for instability in the posterior approach is with the hip in high flexion and gradually increasing internal rotation until subluxation occurs [4, 11]. The vector of the force imparted on the acetabular liner during this movement is typically postero-inferior as internal rotation increases.

Therefore, in the present study, we aim to determine what is the optimal position for placing a lipped acetabular liner in reducing instability for uncemented total hip replacements performed through a posterior approach. We hypothesize that the optimal position may not be in the traditionally assumed postero-superior quadrant.

## Methods

This study was granted ethics approval by the institutional ethical review board (approval number P06/14).

### Validation of smartphone goniometry

In order to obtain objective and accurate measurements of hip positions intraoperatively, we used smartphone accelerometer-based goniometers (SPG). Several studies have shown the usefulness and reliability of this technique in measuring in vivo as well as in vitro joint angles [15–17]. We used freely available apps downloaded from the App Store (“Joint Goniometry”) on two smartphones (Apple, Cupertino, CA, USA) simultaneously and conducted our own validation by comparing it to an established method of determining joint angles using computer-assisted navigation system (“OrthoNav”, Stryker, Kalamazoo, MI, USA). The SPGs were used in the in vivo part of this study because it is less invasive than navigation pins which are required for computer-assisted navigation systems (CAN).

An anatomically accurate sawbone model of the pelvis, left femur, and left tibia was positioned in the lateral decubitus position and stabilized on a table. Navigation pins were inserted into the pelvis and femur as would be in a navigated hip replacement operation. Textiles were wrapped around the sawbones to simulate soft tissue. One smartphone each was attached to the lateral femur and the anterior tibia. The SPGs and CAN were both calibrated in neutral position (0° of hip adduction, flexion, and internal rotation). Flexion angle was gradually increased in increments of 5°, as determined by CAN measurements, until 90° was reached. At each of these increments, SPG readings were recorded, and the whole process from extension to flexion was repeated 10 times.

For internal rotation measurements, the hip was initially placed in neutral adduction/abduction, 90° of flexion and 0° of internal rotation. The femur was then gradually internally rotated in increments of 5° (as determined by CAN) while SPG measurements were recorded. The process was repeated 10 times.

For the purpose of the current study, acceptable error range was defined as 3° on either side of the “gold standard,” i.e., CAN measurements.

### In vivo liner position testing

Fourteen hips in 14 patients were included for the purpose of this study. Age of patients was 67 ± 10 years (range 45–85). Males and females were equally divided. BMI was 28 ± 10. Thirteen patients had a diagnosis of osteoarthritis, and one patient had a primary diagnosis of rheumatoid arthritis.

All patients underwent the same surgical protocol and were operated on by the same surgeon. The operation was performed in the lateral decubitus position using a posterior Kocher-Langenbeck approach. Patients were positioned using a standard pelvic support with two anterior bolsters positioned level at the anterior superior iliac spine (ASIS) and one midline posterior bolster over the sacrum, thereby holding the pelvis vertical on the operating table. Uncemented and hybrid prosthesis (Continuum cup and Avenir / Collarless Polished Taper Stem, Zimmer, Warsaw, IN, USA) were used. Bearing surface for all patients was chrome and cobalt on highly cross-linked polyethylene. Acetabular components were implanted with a target inclination angle of 30–35°, with anteversion to closely match the patient’s native anatomy, using the transverse acetabular ligament as guide. Target femoral component anteversion was 10–15°, with a target combined anteversion of 35–45°. The largest prosthetic femoral heads allowed by the implanted cup were used. Short external rotators were repaired with non-absorbable trans-osseous sutures at the completion of component implantation.

**Fig. 1:**
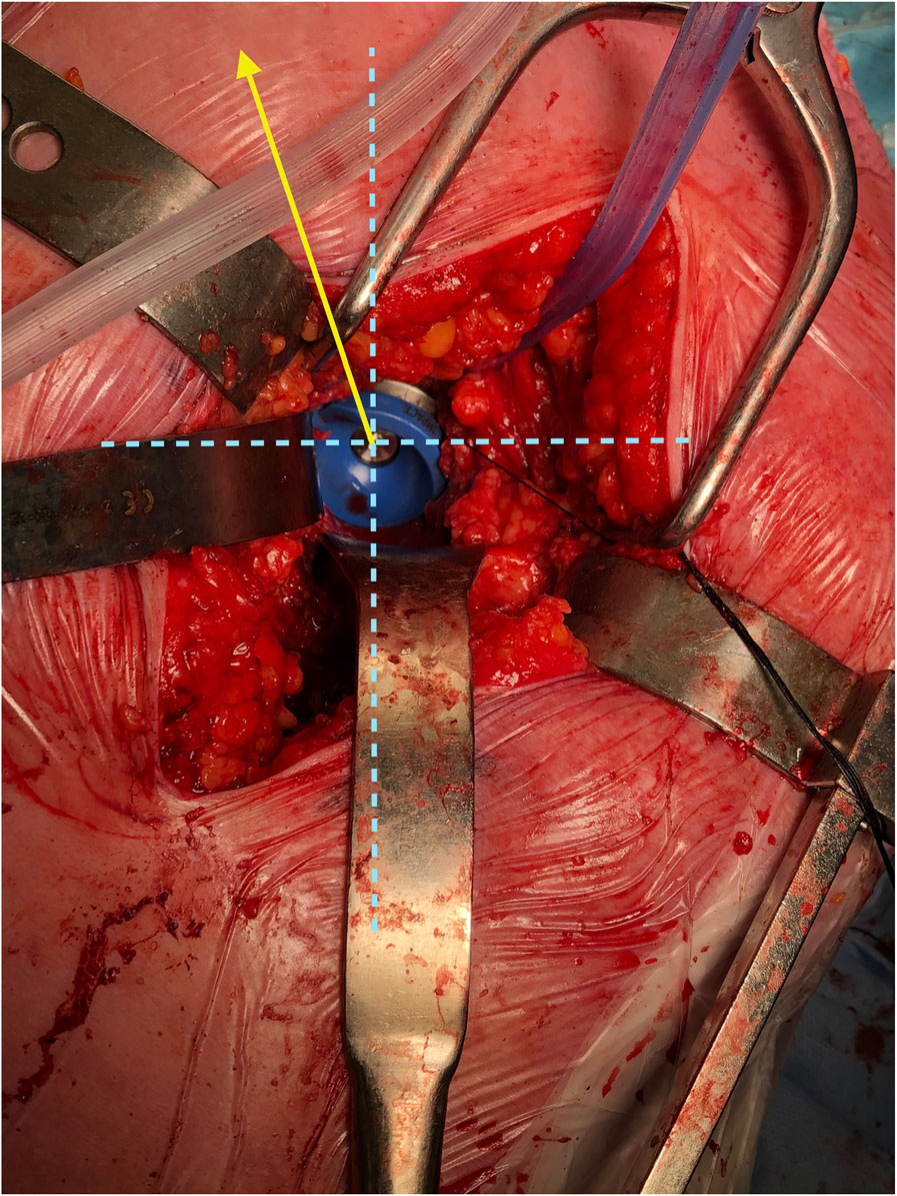
Intraoperative photo demonstrating the left hip with trial liner. Solid yellow line with arrow points to the ASIS and is designated as the 11 o’clock position. Dotted vertical blue line represents the axis of the patient’s body and is the 12 and 6 o’clock reference. The image demonstrates lipped liner being trialed in the posterosuperior quadrant (2 o’clock position)

Hip stability testing was performed using a trial liner with a 10° elevated rim. Both the acetabular shell and liner have 12 notches around its circumference that allowed adjustment of the liner position by increments of 30° or one “hour” on the clock-face. The direction in which the middle of the elevated rim was pointing was designated as the direction of the liner. Twelve o’clock is defined as directly superior, or in line with the patient’s torso and the long axis of the operating table. The ASIS was used as a guide for the antero-superior quadrant (the 1 o’clock or 11 o’clock position, Fig. 1). For each patient, the liner was trialed in seven positions in the posterior half of the clock-face from 12 o’clock to 6 o’clock. A smartphone was placed on the lateral thigh and another on the anterior tibia, both under sterile plastic covering, and secured to the patient with sterile tape. The hip joint was placed in the neutral position, and the SPGs were calibrated for both flexion and internal rotation.

For each liner position, stability was tested in 90° of hip flexion and neutral abduction with gradual increase in internal rotation until subluxation occurred. This is a common method for trialing hip stability intraoperatively and has been reported by others [4, 11]. The maximal amount of internal rotation before subluxation occurred for each liner position was then documented.

Postoperative radiographs were obtained for every patient prior to discharge. Cup inclination angle was measured using the method described by Tannast et al. [18], cup anteversion angle was measured using the method described by Murray [19], and femoral offset was measured using the method described by Lechler et al. [20].

Statistical analyses were performed using Matlab (2017, MathWorks, Natick, MA, USA). Unless otherwise stated, averages are expressed as mean ± standard deviation.

## Results

### Smartphone goniometry validation

**Fig. 2:**
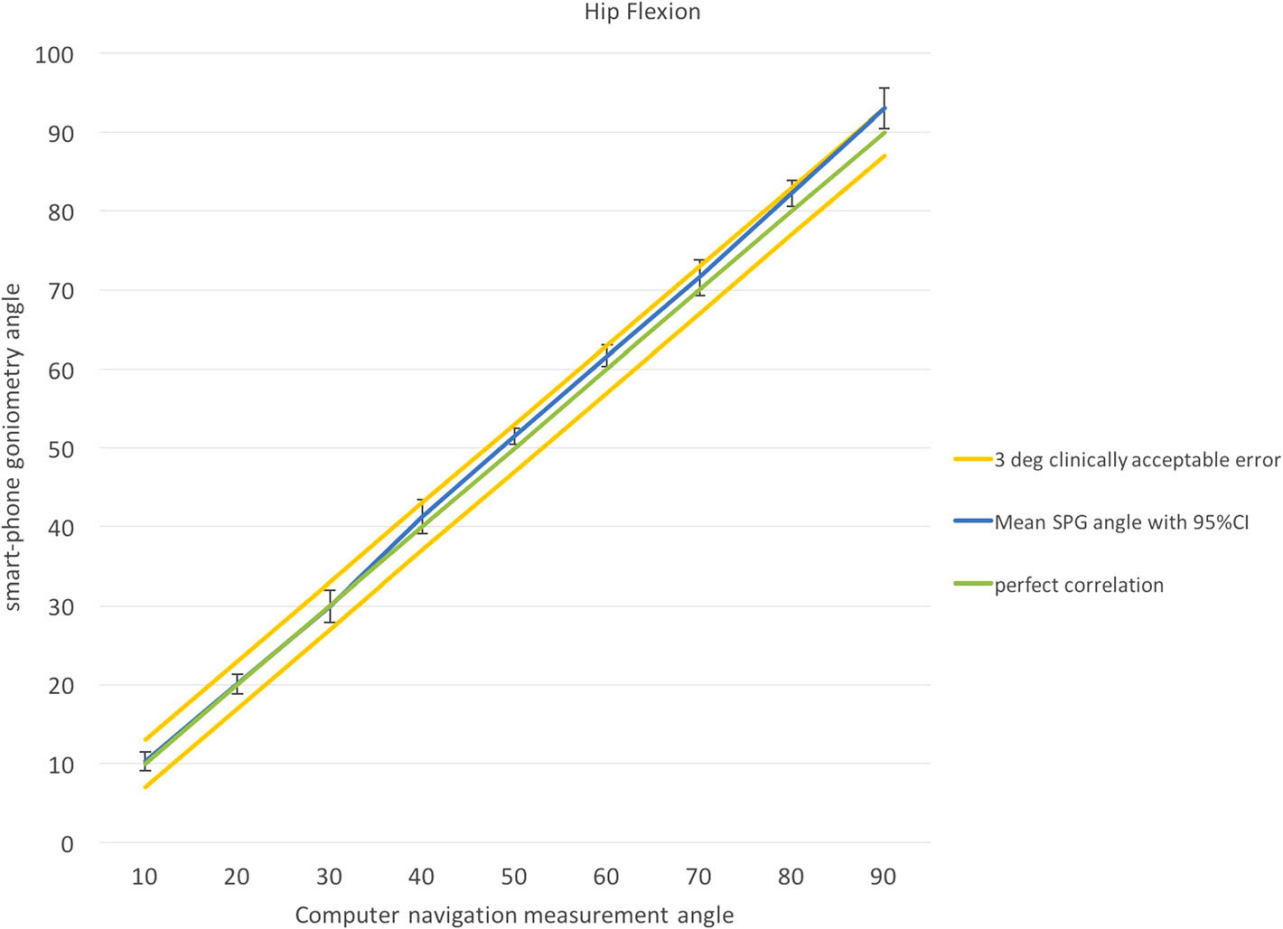
Correlation of smartphone goniometer reading with computer-assisted navigation for hip flexion. At all joint angles measured, mean smartphone readings were within a 3° clinically acceptable error range. SPG: smartphone goniometer

**Fig. 3:**
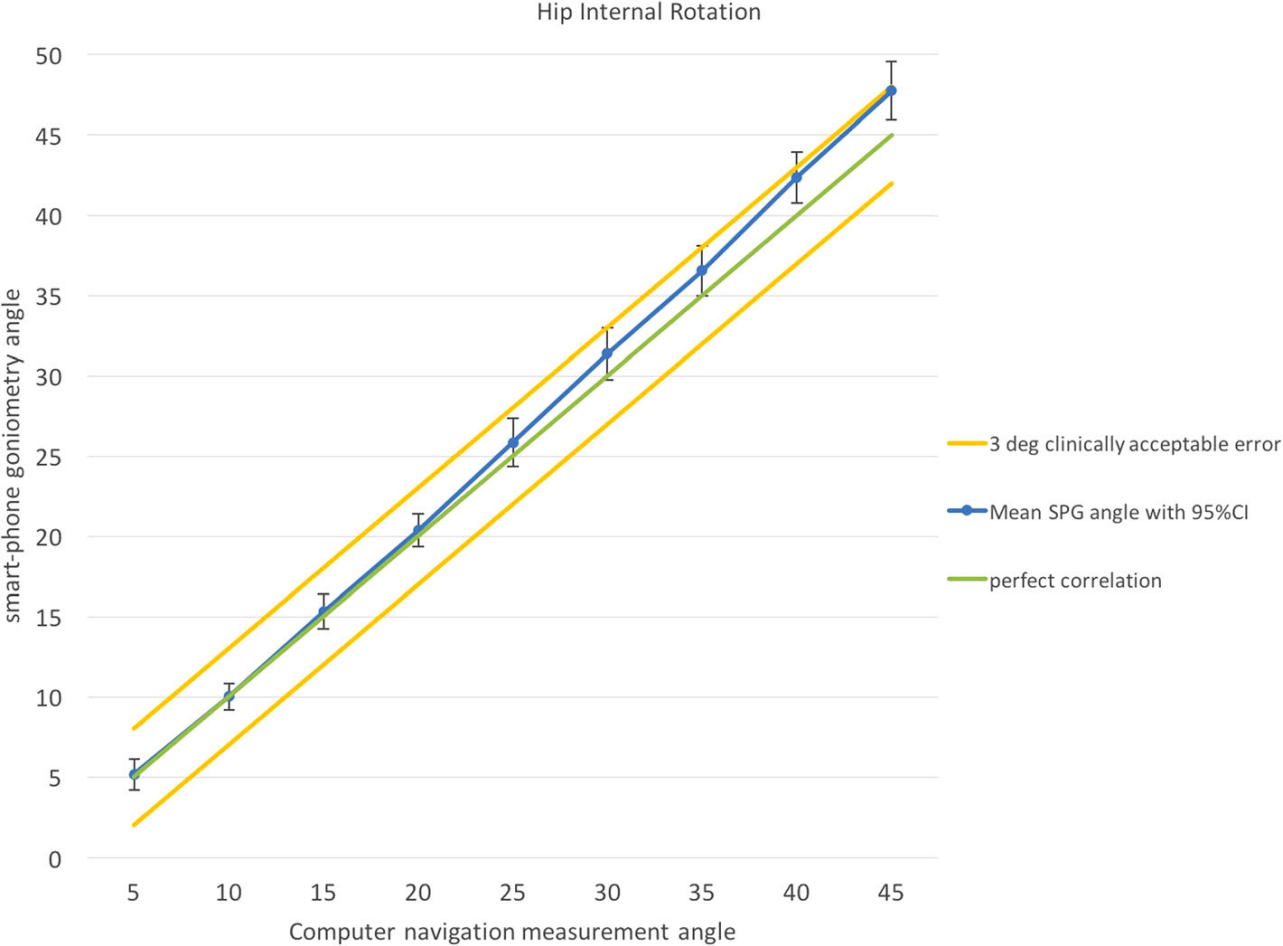
Correlation of smartphone goniometer reading with computer-assisted navigation for hip internal rotation at 80° hip flexion. At all angles measured, mean smartphone readings were within a 3° clinically acceptable error range. SPG: smartphone goniometer

Good correlation can be seen between CAN and SPG measurements as shown in Figs. 2 and 3. For all joint angles, the SPG falls within the clinically acceptable range. SPG measurements were remarkably consistent, with the standard error of the mean being less than 0.5° for all joint angles measured.

### In vivo testing

Median acetabular shell size used was 58 mm diameter (range 50–62 mm). Six 36-mm and eight 32-mm femoral head components were used.

**Fig. 4:**
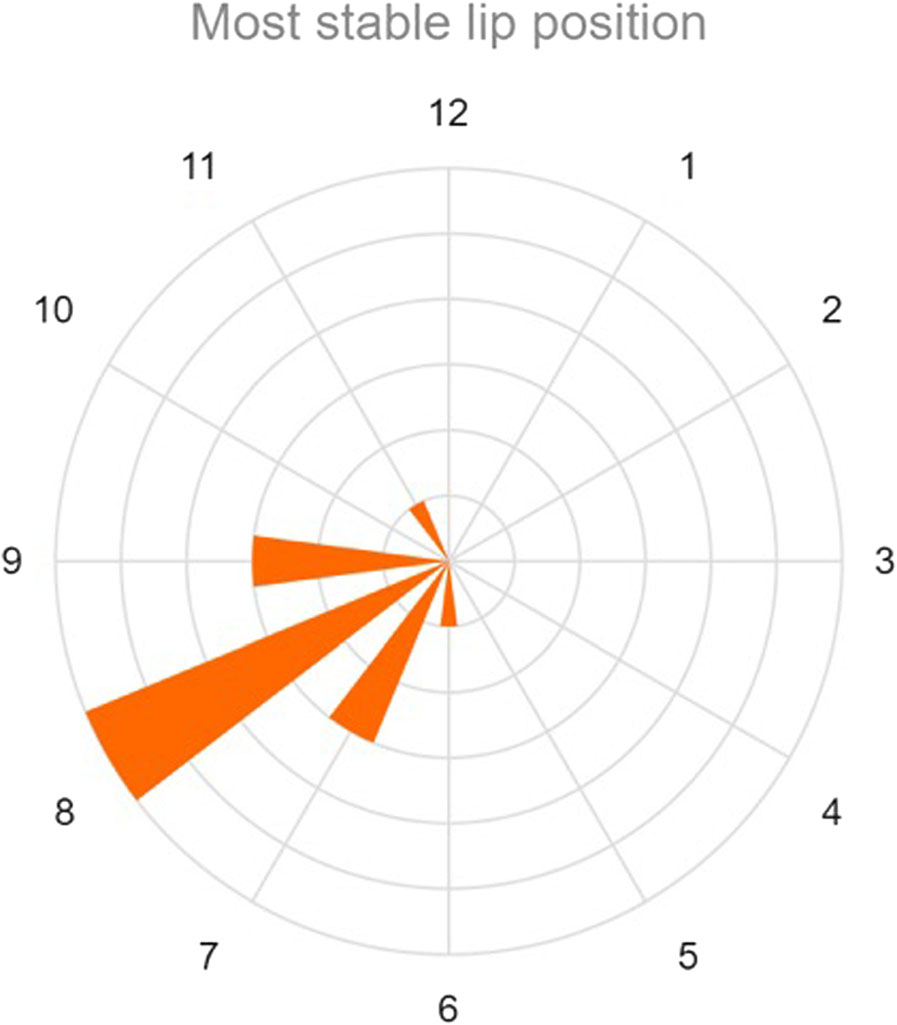
Most stable lip position for elevated liners (transcribed for right hip). Length of the clock “hands” represent number of patients. The most common position tested that imparted greatest stability in each hip was at the 8 o’clock position, i.e., in the postero-inferior quadrant

As seen in Fig. 4, the most common stable position for the lip of the liner was in the postero-inferior quadrant (8 o’clock for right hip, 4 o’clock for left hip). The equal second most common stable positions were 7 o’clock and 9 o’clock (5 o’clock and 3 o’clock for left hip). Converting this to degrees, the average for the most stable position was at 115° (direct superior = 0°, direct posterior = 90°).

The range for most stable liner position for different patients varied from postero-superior (11 o’clock/1 o’clock position) to directly inferior (6 o’clock position).

**Fig. 5:**
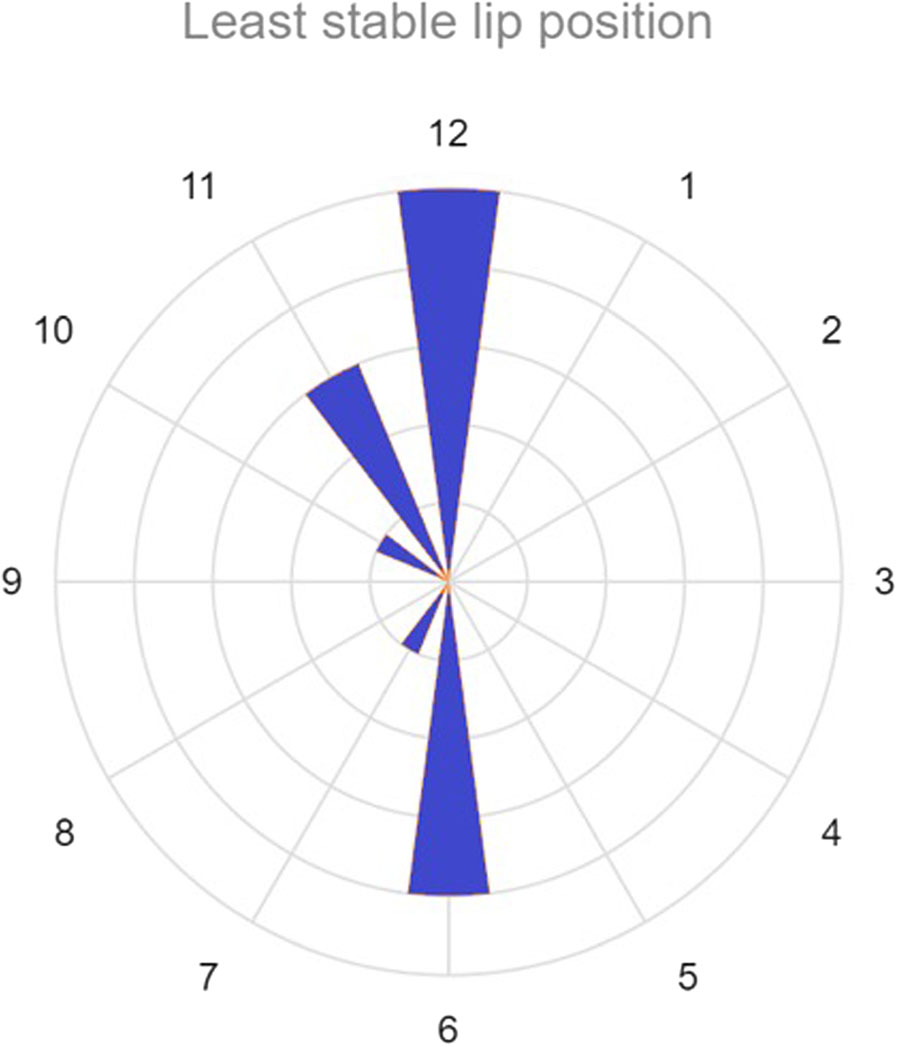
Least stable lip position for elevated liners (transcribed for right hip). Length of the clock “hands” represent number of patients. The most common position tested that imparted least stability in each hip was at the 12 o’clock position (i.e., directly superior)

Of the liner positions measured, the most common lip position that conferred the least stability was at 12 o’clock (Fig. 5). This was followed by the 6 o’clock position.

Comparing a non-lipped liner to a lipped liner placed in the optimal position, the average difference in internal rotation gained prior to occurrence of subluxation was 23° (*p* = 0.003).

**Fig. 6:**
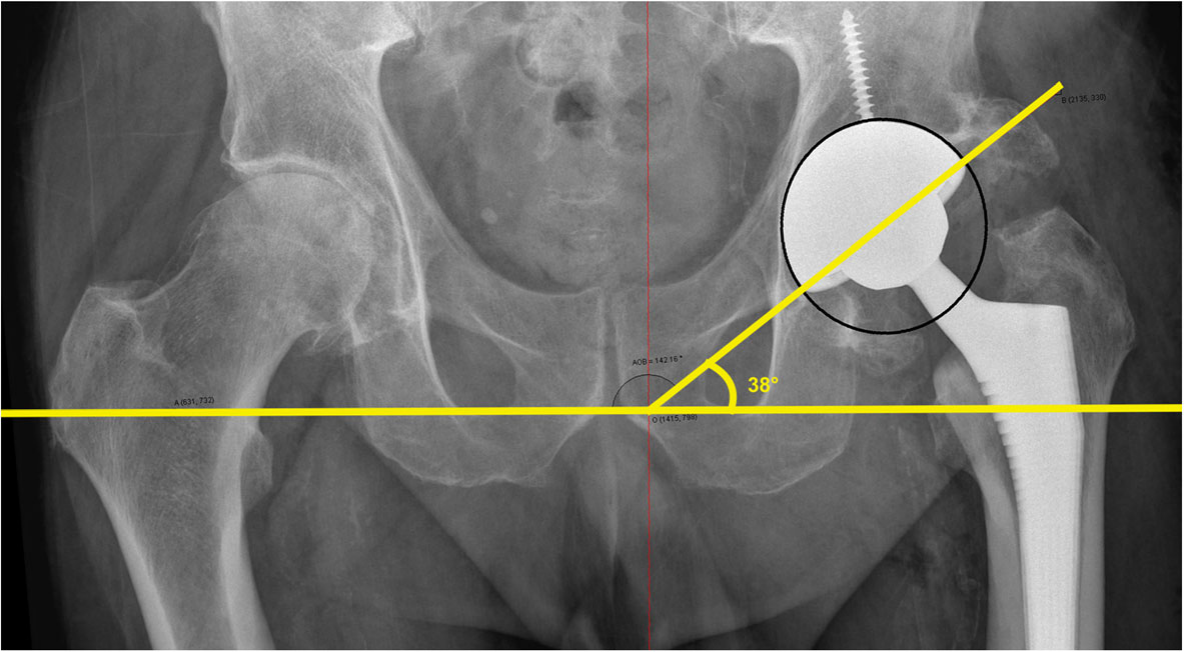
Post-operative antero-posterior radiograph of the pelvis with measurements of cup inclination. This particular cup measured 38° inclination

As measured on post-operative radiographs, cup inclination angle was 31° ± 6° (Fig. 6), and cup anteversion angle was 17° ± 5°. Post-operative femoral offset measured 105 ± 5% of the contralateral side.

There was no significant association found between most stable liner position and cup inclination (*R*^2^ = 0.09, *p* > 0.05) or anteversion angles (*R*^2^ = 0.11, *p* > 0.05). Three hips had cup inclination angle of > 35° (36°, 38°, and 41°). Of these, all three demonstrated most stable liner position in the postero-inferior quadrant. Results were also analyzed with head sizes separated into 36 mm (*N* = 6) and 32 mm (*N* = 8) diameters. The most common optimal liner position for 36-mm heads was the 8/4 o’clock position (*N* = 3, 50%), with the second most common being the 3/9 o’clock position (*N* = 2, 33%). The most common optimal liner position for 36-mm heads was also the 8/4 o’clock position (*N* = 3, 38%), with the second most common being the 5/7 o’clock position (*N* = 2, 25%).

## Discussion

Lipped or elevated acetabular liners are frequently used in total hip arthroplasty to improve stability. Our results show that lipped liners placed in the postero-inferior quadrant appeared to allow more internal rotation prior to subluxation and hence increased stability, when compared to those placed in the postero-superior quadrant.

It is unclear how the prevailing practice of placing the lip of the liner in the postero-superior quadrant was first established. A search of the literature reveals no supporting evidence. The practice was first introduced by Charnley in 1972 [6], when he modified the scalloped liner into one with a long postero-superior wall. The surgical approach used by Charnley and his peers then was mainly the trans-trochanteric approach, and dislocations were frequently associated with non-union or displacement of the trochanteric osteotomy. In addition, prosthetic femoral head sizes were much smaller at 22 mm than today’s femoral heads, while poorer quality polyethylene meant greater liner wear particularly in the superior portion. We postulate that the mechanics of instability in that setting may be different to those seen in the modern total hip arthroplasty performed through a posterior approach. These factors may have influenced early surgeons in placing lipped liners in the postero-superior quadrant, a practice that has continued to this day.

Sultan et al. [11] tested the effect on stability of using a lipped liner intraoperatively and compared it with a non-lipped liner. All liners were tested in the postero-superior quadrant in the 10 or 2 o’clock position. Their method of testing was similar to the present study, with the hips being placed into high flexion and then gradually internally rotated until subluxation occurred. However, measurements were performed using a traditional goniometer which can give rise to inaccuracies especially in the intraoperative setting. In the present study, liner position was tested in multiple positions and measurements were performed more objectively with the use of a validated smartphone goniometer.

Sultan et al. found that when all else was equal, compared to a neutral liner, using a lipped liner at the 10 or 2 o’clock position resulted in an increase of 8° in the amount of internal rotation needed to cause posterior dislocation. Our results show that when comparing neutral liner to an optimally placed lipped liner resulted in an increase of 23° in the amount of internal rotation needed to cause subluxation. One possible explanation for this difference is that an optimally placed lipped liner may confer further stability than a lipped liner placed routinely in the postero-superior quadrant.

Use of a lipped liner is not without its controversy. Some authors have raised concerns that it can reduce range of motion and cause impingement, increased polyethylene wear debris, or levering and dislocation in the opposite direction to the elevated lip [21, 22]. However, strong evidence exist that by far most dislocations in hips performed through a posterior approach occur in the posterior direction and any potential disadvantages of a lipped liner is offset by the reduced risk of posterior dislocations associated with its use [5, 7–9].

We believe one strength of this study is that it is an in vivo study. Although many other factors (such as femoral version, acetabular version, head-neck ratio) can contribute to hip instability, by testing multiple liner positions in each individual patient after implantation of the acetabular and femoral components, these potentially confounding factors are inherently controlled for. The use of a validated SPG system also removes much of the inaccuracies of measuring joint angles intraoperatively.

We acknowledge weaknesses to this study. While hip flexion and internal rotation can be measured with relative accuracy using the SPG, liner position in the acetabular shell may not be as accurate. We used the line of the patient’s body, axis of the operating table, and the anterior superior iliac spine (ASIS) as a guide to the position of the liner on the clock-face. Ultimately, our study hopes to help other surgeons decide where to place the lipped liner intraoperatively, and in most cases, these visual cues are the only intraoperative guides available in determining what the acetabular orientation is. Secondly, although we measured postoperative cup inclination, anteversion, and femoral offset radiographically, we did not perform routine postoperative computed-tomography or other three-dimensional studies to allow accurate measurement of femoral anteversion and leg length. Thirdly, two different femoral head sizes (32 mm and 36 mm) were used, potentially introducing a new confounding variable. Finally, the average cup inclination angle in our series (31° ± 6°) are on the lower side of what is usually aimed for, as recommended by Lewinnek et al. [23]. It is possible that optimal liner position may differ if cup inclination was lower. However, within the range of cup inclination and anteversion angles in our series, we did not detect any correlation with optimal liner position.

## Conclusion

When tested intraoperatively, a lipped liner placed in the postero-inferior quadrant (8 o’clock for right hip or 4 o’clock for left hip) is more likely to confer greater stability against posterior dislocation in total hip replacements performed through a posterior approach. This appears to hold true in our cohort where the average cup inclination is 31°. However, the results may not be generalizable for higher cup inclination angles.
